# The Accumulation and Metabolism Characteristics of Rare Earth Elements in Sprague–Dawley Rats

**DOI:** 10.3390/ijerph17041399

**Published:** 2020-02-21

**Authors:** Bing Cao, Jing Wu, Changlian Xu, Yan Chen, Qing Xie, Li Ouyang, Jingyu Wang

**Affiliations:** 1 School of Psychology and Key Laboratory of Cognition and Personality (Ministry of Education); National Demonstration Center for Experimental Psychology Education, Southwest University, Chongqing 400715, China; bingcao@swu.edu.cn; 2 Medical and Health Analysis Center, Peking University, Beijing 100191, China; wujingj@126.com (J.W.); Xieqing94@bjmu.edu.cn (Q.X.); ouyangli@bjmu.edu.cn (L.O.); 3 College of Environmental Science, Sichuan Agricultural University, Chengdu 611130, China; chinaxuchanglian@126.com; 4 Dalla Lana School of Public Health, University of Toronto, Toronto, ON M5T 3M7, Canada; yann.chen@mail.utoronto.ca; 5 Department of Laboratorial Science and Technology, School of Public Health, Peking University, Beijing 100191, China; 6 Vaccine Research Center, School of Public Health, Peking University, Beijing 100191, China

**Keywords:** rare earth elements, metabolism, SD rats, inductively coupled plasma-mass spectrometry

## Abstract

The current study aims to investigate the influence of five rare earth elements (REEs) (i.e., lanthanum (La), cerium (Ce), praseodymium (Pr), neodymium (Nd), and gadolinium (Gd)) on the growth of Sprague–Dawley (SD) rats, and to explore the accumulation characteristics of REEs in tissues and organs with different doses as well as the detoxification and elimination of high-dose REEs. Fifty healthy male SD rats (140~160 g) were randomly divided into five groups and four of them were given gavage of sodium citrate solution with REEs in different doses, one of which was the control group. Hair, blood, and bone samples along with specific viscera tissue samples from the spleen and the liver were collected for detection of REEs by Inductively Coupled Plasma-Mass Spectrometry (ICP-MS). Treated rats expressed higher concentrations of REEs in the bones, the liver, and spleen samples than the control group (*P* < 0.05). Few differences were found in relative abundance of La, Ce, Pr, Nd, and Gd in the hair and the liver samples, although different administration doses were given. The relative abundance of Ce in bone samples was significantly lower in the low-dose group and control group, whereas the relative abundance of La and Pr in the bone samples were highest among all groups. Although in the REEs solution, which was given to rats in high-dose group, the La element had a higher relative abundance than Ce element, it ended up with higher Ce element relative abundance than La element in the spleen samples. REEs had a hormetic effect on body weight gain of SD rats. The accumulation of the measured REEs were reversible to low concentrations in the blood and hair, but non-reversible in the bones, the spleen, and the liver. Different tissues and organs can selectively absorb and accumulate REEs. Further inter-disciplinary studies about REEs are urgently needed to identify their toxic effects on both ecosystems and organisms.

## 1. Introduction

With annual increasing amounts exploited and diversity of uses explored, rare earth elements (REEs) are extensively applied to industrial, medical, zootechnical, and agricultural fields [1,2]. The REEs are not as rare in geological abundance as other toxic metals, and China has around 90% of the world’s detected REEs [3,4]. The expanding applications of REEs have led to a great deal of human exposures from occupational, environmental, medical, and iatrogenic routes [5]. Since REEs were commonly incorporated into agriculture, forage additives, and fertilizers [2,6], they can be up-taken, bio-accumulated, and bio-magnified by the human body via the food chain [7], and accumulate in the human body to the ends of life after they enter the body [8]; thus, more concerns and further investigations about the long-term effects of REE accumulations on human health are urgently needed. 

Extensive research has proven that REE exposures would increase concentrations of REEs in body organs and tissues [9,10]. The REE accumulations were also observed in various organs of human beings who were exposed, long term, to REE particles [6]. A Chinese study of children who resided in REE mining areas reported that REE concentrations in the scalp/hair samples were inversely proportional to the distance of their residence from mining areas [11]. Moreover, workers in industries such as photoengravers, glass or lens polishers, and movie projectionists who undergo long-term occupational exposures to REE particles have a higher risk to suffer rare earth pneumoconiosis, compared to the control group without REE exposures [6,12]. What is more, the REE-induced tissue-specific bioaccumulations have also been reported. Compared to the control group, higher REEs that accumulated in multiple tissues and organs, including the lung, the liver, the kidneys, and nails, were found in the population with REE exposures [13]. In addition, excessive exposure to REEs could also impact the intelligence quotients (IQs) of children, showed by studies of children who live in high La element background regions and attained lower IQ scores than those children in non-exposed regions [14]. Moreover, metabolism and toxicity of REEs have been extensively investigated by oral, intravenous, and intraperitoneal administration, as well as instillation or intratracheal instillation in various animal models [15]. A large number of biological events could cause the re-distribution of REEs, but the re-distribution process is neither well-defined nor explored. Even now, less attention has been paid to the re-distribution and accumulation processes of REEs in different organs and tissues.

Taken together, the mechanism and the relevance of REEs to health and disease in human body remain obscure. Rats are standard experimental animals for toxicological research for chemicals and are frequently used to observe the toxicological effects of trace elements [16,17,18]. Herein, the main purpose of the current study is to explore the accumulation characteristics of selected REEs in different tissues and organs, as well as the detoxification and elimination of high-dose REEs of Sprague–Dawley (SD) rats. We selected the five most commonly used elements in agriculture and industry for our current experiment, including four different light REEs (i.e., lanthanum (La), cerium (Ce), praseodymium (Pr), and neodymium (Nd)) and one heavy REE (i.e., gadolinium (Gd)), in order to explore the re-distribution and accumulation patterns with different exposure doses. In our belief, the findings presented in the current study will provide a more comprehensive understanding of REEs’ re-distribution pattern from the environment to human body as well as perceiving the effects of the selected REEs on health and disease.

## 2. Materials and Methods 

### 2.1. Animal Experiment and Grouping

All animal care and experimental procedures involved in the current study conformed to the Guide for the Care and Use of Laboratory Animals. This study was approved by the Committee on the Ethics of Animal Experiments of the Peking University Health Science Center.

Fifty physically healthy male SD rats, weighing 140~160 g, were involved in this study. The SD rats were obtained from the Department of Laboratory Animal Science, Peking University Health Science Center, and maintained in a specific pathogen-free (SPF) laboratory with controlled temperature (24 ± 1 ℃), relative air humidity (60%), and 12-h light and dark cycles. The SPF grade hardwood chip bedding and SPF grade food were also obtained from the Department of Laboratory Animal Science, Peking University Health Science Center. All animals were housed in individually ventilated cages covered with hardwood chip bedding and were given free access to sufficient SPF grade maintenance diet and water ad libitum. After a week of adaptive feeding, rats were all shaved by electric hair clipper (POVOS brand, Beijing Biorj Technology Co., Beijing, China) to prevent additional particle insertion.

The rats were randomly divided into five groups: The control group, low-dose group, medium-dose group, high-dose group, and detoxification group, with 10 rats in each group (n = 10). Among the rare earth citrate used in our experiments, La element possessed 6.9% of the solution, Ce: 15.4%, Pr: 1.7%, Nd: 1.1%, Gd: 0.22%, and other REEs: 0.38%. The REE solution, prepared with rare earths citrate, was diluted with pure water to different dose groups (i.e., 5 mg/mL, 50 mg/mL, 500 mg/mL for low-, medium-, and high-dose groups, respectively). The rats in the control group were prepared with pure water and sodium citrate to eliminate the effect of [Cit]^3−^ on results. The administration dose of REEs in each group was 0, 50, 500, and 5000 mg/(kg bw), respectively. For the solution used in the control group, we diluted the sodium citrate with pure water until it reached a concentration of 35%. The contents of REEs in the food and the rare earth citrate are shown in Supplementary Table S1. All rats were given treatments by gavage. The frequency of gavage was once a day for 4 weeks, and the amount of gavage was increased simultaneously with the increase of body weight (i.e., always 1.0 mL gavage solution per 100 g of body weight). The body weights of rats were measured 6 times in total, including the day of enrollment (1 time), the beginning of every experiment week (4 times), and the day of execution (1 time). 

### 2.2. Sample Procedures 

After five weeks (including a week of adaptive feeding and four weeks of gavage), all groups except for the detoxification group (i.e., control group, low-dose group, medium-dose group, high-dose group, n = 40) were anaesthetized by intra-peritoneal injection of 10% chloral hydrate (3 mL/kg) and were then decapitated. The blood samples were collected from the femoral artery. We anticoagulated the blood samples with heparin sodium, centrifuged at a speed of 2000 r/min for 10 min. The bones, livers, and spleens of SD rats were also collected. Rats in detoxification groups (n=10) discontinued gavage with REEs and then continued to feed for 4 weeks in the same environment. The samples from the detoxification group were collected with the same protocols as other groups after four weeks of drug withdrawal. All the samples were washed with deionized water three times and wiped with filter paper to collect residual water, and then stored at -70 ℃ until use. 

### 2.3. Sample Pretreatment and Determination 

The tissue samples were washed with the distilled water. After being dried with clean gauze, the tissue samples were triturated and weighed accurately (i.e., whole blood: 0.2 mL, spleen: 0.2 g, hair: 0.2 g, liver: 0.2 g, bone, 0.1 g). The samples used for determination of REEs were digested by wet method. The outline of wet digestion is as follows: After being weighed, the samples were digested by HNO_3−_HClO_4_ mixture and evaporated to near dryness, transferred to 10 mL-volumetric flasks, and then diluted with deionized water to volume, and mixed.

The concentration of REEs in different organs was determined by Inductively Coupled Plasma-Mass Spectrometry (ICP-MS, Pekin-Elmer Elan6000-DRCⅡ, Perkin-Elmer Instruments, Waltham, MA, USA). The measured and referred concentrations of the five REEs for the standard references (GBW09101a, National Standard Material Center in China) were used for quality control. All the determination and quantitative analyses were conducted in the Central Laboratory of Biological Elements in the Peking University Health Science Center, which have been qualified by China Metrology Accreditation (CMA) system.

### 2.4. Statistical Analysis

Statistical analyses were performed by using SAS 9.1.3 (SAS Institute, Cary, NC, USA). The mean and standard deviation were used to describe the concentrations of trace elements in different groups. Relative abundance was calculated by dividing one REE concentration by the sum of all detected REE concentrations. The geometric mean was used to describe how many folds of the trace elements had been increased in low/medium/high-dose groups compared to the control group. We compared differences among the groups by using one-way ANOVA. Spearman’s rank correlation was used to determine the correlations of the REE concentrations among different tissues and organs of rats. Pair-wise comparison was performed by Student-Newman-Keuls (SNK) test, and a two-sided *P*-value < 0.05 was considered statistically significant.

## 3. Results

### 3.1. Body Weight

**Figure 1:**
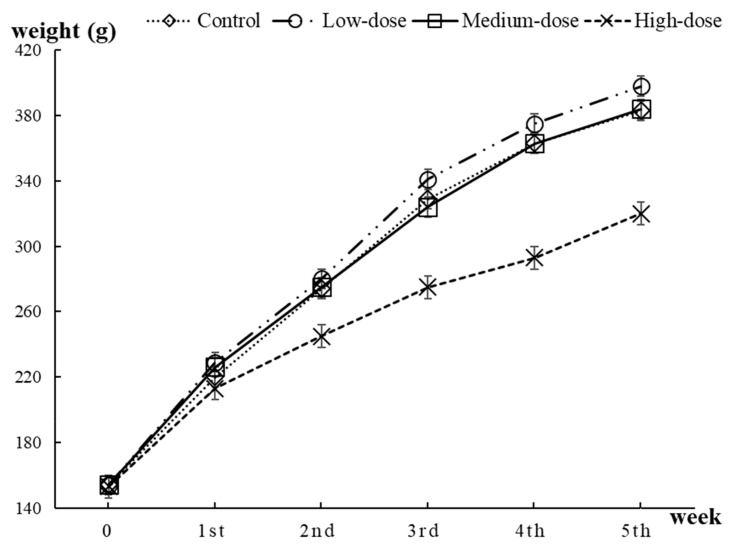
Body weight of Sprague–Dawley (SD) rats with different doses of rare earth elements (REEs).

In total, 50 rats were included in the body weight analysis. After a week of adaptive feeding and four weeks of gavage feeding, weight gained by rats was positively correlated to the increase of feeding time. The average body weight of rats in low-dose group was the highest while rats in the high-dose group had the lowest body weight among the all groups (i.e., control group: 383 ± 22 g, low-dose group: 398 ± 13 g, medium-dose group: 384 ± 20 g, high-dose group: 320 ± 24 g), the difference was statistically significant (*P* < 0.05). The details of body weight are shown in Figure 1.

### 3.2. REE Concentrations in Different Tissues and Organs

The absolute concentrations of five REEs in different tissues and organs of SD rats are summarized in Table 1. The REE (i.e., La, Ce, Pr, Nd, and Gd) concentrations in the organ and tissue samples were all highest in the high-dose group and lowest in the low-dose group. In the blood samples, no statistical differences were observed in the concentrations of five REEs among all groups (*P* > 0.05) except for the high-dose group. In the liver, the spleen, and the bone samples, all five REEs were found significantly higher in the detoxification group than that in the control, low-dose, and medium-dose groups (*P* < 0.05). 

The results of relative abundance analysis (Table 2) indicated that blood samples of rats in the high-dose group contained similar relative abundance of Nd and Gd elements to test substances that were originally given to them (Nd: 3.48 ± 0.96% vs. 4.28%; Gd: 0.95 ± 0.12% vs. 0.86%), and lower relative abundance than other groups. Moreover, compared to the test substances (Ce: 59.92%), the relative abundance of Ce element was lower in bone samples in the control group (17.49 ± 5.15%) and the low-dose group (21.12 ± 6.15%). However, the relative abundance of La and Pr elements had the opposite traits of Ce element in the bone samples. What is more, in the spleen samples, the relative abundance of La and Ce elements were higher in the high-dose group than in the test substances (La: 81.29 ± 1.19% vs. 26.85%; Ce: 10.62 ± 0.18 vs. 59.92%). In addition, the relative element abundance of La, Ce, Pr, Nd, and Gd did not change much between test substances and different administration dosages in the hair and the liver samples.

### 3.3. Correlations among Different Tissues and Organs of Rats

The correlations of the five REE concentrations among different tissues and organs are shown in Table 3. The correlation coefficients of La, Ce, and Pr elements were high between the bones and the liver (La: 0.849, Ce: 0.877, Pr: −0.817), as well as hair and the bones (La: −0.791, Ce: −0.760, Pr: 0.790). There were negative correlations of La, Ce, and Pr elements between hair and the liver (La: −0.680; Ce: −0.745; Pr: −0.822). In addition, there was no correlation between whole blood and any other organs or tissues, and neither did any correlation exist between the spleen and other organs or tissues, since their absolute values of correlation coefficients were all lower than 0.600.

## 4. Discussion

The re-distribution patterns of the five REEs were different among the tissues and organs of SD rats in the presence of exogenous factors. The REEs stimulated body weight gain of SD rats in the low-dose group and restrained in the high-dose group. From the findings of the current study, we can conclude that five REEs, typically La, Ce, Pr, Nd, and Gd elements, had a hormetic effect on body weight gain of SD rats. The weight gain can be interpreted as our engine body having an adaptive response to the weak stimuli of REEs. Calabrese et al. summarized that hormesis has commonly happened in thousands of situations [19,20,21]. Previous evidence also assumed that a small amount of REEs in food could increase body weight in various animals, including pigs, chicken, fish, rabbits, etc. [6]. A study of 90-day, repeated oral gavage of La element in male rats reported the weights of the liver, spleen, kidney, heart, and thymus in the high-dose group (i.e., 144.0 mg/kg BW) were significantly lower than those in the controls, but did not report that La element stimulated body weight gain of SD rats in the low-dose group [22].

After four weeks of gavage, REEs were primarily localized in various organs. In this experiment, we found that the order of the REEs concentration from highest to lowest to be hair, the liver, the spleen, bones, and blood. It was shown that the distribution patterns of REEs were similar to those of other heavy metals, which was in agreement with the previous research. The result from the present research also indicated that the REEs can be enriched in the hair [23,24].

The analysis of absolute content analysis indicated that the concentrations of five REEs in rats’ tissues and organs were proportional with the administration doses. The current study was unable to find any statistical difference of La, Ce, Pr, Nd, or Gd contents among detoxification groups, control group, and low-dose group in the whole blood samples and hair sample. However, the detoxification group had higher REE contents than the control group and low-dose group in the bones, the liver, and the spleen. These results were consistent with research findings of Y. Nakamura [23], both of the two studies indicating that bones, the liver, and the spleen had poor metabolism of La, Ce, Pr, Nd, and Gd elements. Another study, which is on retention of Gd following linear gadolinium-based contrast agent administration in rats, also supported our findings. The study found that even if Gd clears rapidly in the circulation system, the brain and bones still retained substantial Gd after detoxification for six weeks [17]. A study of 30-day repeated oral administration of lanthanum oxide nanoparticles on elements in the bones of Wistar rats also supported our findings, where lanthanum oxide nanoparticles were incorporated on the surface of the bones [18]. An experiment of Wistar rats that were intraperitoneally exposed to soluble solution of La and Ce elements for two weeks demonstrated the toxicity of these elements with doses used [25]. 

In this study, the relative element abundance of La, Ce, Pr, Nd, and Gd elements changed significantly in the whole blood, bones, and the spleen, while they remained relatively stable in the liver and hair. This scene may indicate that La, Ce, Pr, Nd, and Gd elements were able to be selectively accumulate in the whole blood, bones, and the spleen rather than in the hair and the liver. The distribution pattern of REEs in the low-dose group was similar to that in the control group, which indicated that when the supplements were under the threshold, the body would follow the physiological need to absorb the REEs. When the concentrations of REE dosages exceeded the threshold, the accumulation would be compelled to higher levels, where the distribution patterns differed from that of the low-dose and control groups. The results were also supported by some other experiments: Zhang et al. reported that when the level of REEs were under the threshold, it could promote the UMR106 hyperplasia and differentiation. These effects could not be observed with higher concentration of REEs [24]. The fact that the relative element abundance changes in whole blood, bones, and the spleen tissues can reflect the status of these five REEs in the real world. In addition, whole blood is considered to be a good biomarker for some trace elements by reflecting moderate degree of REEs that people commonly get exposed in modern daily lives [26,27]. Different REEs might have different target organs, and Kim et al. found that the target organ of neodymium oxide (NdO) in rats was determined to be the lung [28]. Considering the correlations and significance of changes from exogenous REE exposures, the liver may be the best choice for evaluating the effects of La, Ce, and Pr elements for exogenous exposures. 

This study directly reported the effects of the selected REEs on rats’ body weight and the re-distribution patterns of those REEs from the environment to rats. Our current findings herein provided valuable evidence for the toxicity evaluation and risk assessment of REEs, understanding the re-distribution patterns of REEs from the environment to human body, as well as REEs control strategies. The current study has to be interpreted in the light of come limitations. First, the relatively small sample size may limit the extrapolation of the current research results. Second, total REE concentrations were measured without specificity regarding the specific form and/or valence state of the metal. Third, we failed to detect other REEs in the current study, which should also be affected by the intake of selected REEs. Fourth, the length of the tibia should be one of the parameters to evaluate the real impact of REEs on rats’ growth, in addition to body weight gain. We did not collect the data of the length of the tibia in the current study due to limited time and resources. In addition, throughout the trial, placing the animals in metabolic cages and analyzing the concentrations of REEs in the urine and feces could provide more comprehensive and accurate evidence. Further inter-disciplinary studies about REEs are urgently needed to identify their toxic effects on both ecosystems and organisms.

## 5. Conclusions

In conclusion, the REEs, particularly La, Ce, Pr, Nd, and Gd elements, had a hormetic effect on body weight gain of SD rats. The accumulation of La, Ce, Pr, Nd, and Gd elements is reversible to low concentrations in the subjects’ whole blood and hair, but not in the bones, spleen, and liver. Different parts of tissues and organs can absorb and accumulate REEs selectively, especially the bones and the spleen because they have a poor capacity for accumulation of Ce element. Our current findings herein provided valuable evidence for the toxicity evaluation and risk assessments of REEs. The accumulation characteristics, metabolism, and function of REEs in the bones, the spleen, and the liver should be further explored. 

## Figures and Tables

**Table 1:** The absolute concentration of the five REEs in the different tissues and organs from rats in the different groups.

		Control (n=10)	Low-Dose (n=10)	Medium-Dose (n=10)	High-Dose (n=10)	Detoxification (n=10)	*P-Value*
Whole Blood (ng/mL)							
	La	2.71 ± 1.56 ^d^	3.12 ± 1.84 ^d^	2.28 ± 0.77 ^d^	37.2 ± 21.6 ^a,b,c,e^	2.03 ± 0.91 ^d^	<0.001
	Ce	4.89 ± 2.87 ^d^	5.95 ± 3.56 ^d^	4.47 ± 1.41 ^d^	76.76 ± 43.89 ^a,b,c,e^	4.21 ± 1.85 ^d^	<0.001
	Pr	0.55 ± 0.33 ^d^	0.67 ± 0.39 ^d^	0.49 ± 0.15 ^d^	8.47 ± 4.86 ^a,b,c,e^	0.39 ± 0.21 ^d^	<0.001
	Nd	0.99 ± 0.29 ^d^	1.17 ± 0.42 ^d^	0.9 ± 0.17 ^d^	4.34 ± 2.29 ^a,b,c,e^	0.91 ± 0.45 ^d^	<0.001
	Gd	0.2 ± 0.05 ^d^	0.24 ± 0.08 ^d^	0.18 ± 0.01 ^d^	1.16 ± 0.54 ^a,b,c,e^	0.17 ± 0.07 ^d^	<0.001
Spleen (ng/g)							
	La	2.22 ± 1.34 ^d,e^	2.71 ± 1.00 ^d,e^	68.96 ± 35.73 ^d,e^	10,559.94 ± 8994.95 ^a,b,c,e^	778.15 ± 588.96 ^a,b,c,d^	<0.001
	Ce	2.51 ± 2.68 ^d,e^	3.22 ± 2.24 ^d,e^	158.86 ± 77.19 ^d,e^	1382.2 ± 1187.87 ^a,b,c^	2264.89 ± 1495.49 ^a,b,c^	<0.001
	Pr	0.47 ± 0.3 ^d,e^	0.6 ± 0.29 ^d,e^	20.74 ± 9.84 ^d,e^	570.17 ± 520.3 ^a,b,c,e^	297.31 ± 195.57 ^a,b,c,d^	<0.001
	Nd	0.3 ± 0.28 ^d,e^	0.62 ± 0.28 ^d,e^	8.27 ± 3.19 ^d,e^	430.29 ± 404.95 ^a,b,c,e^	119.69 ± 89.2 ^a,b,c,d^	<0.001
	Gd	0.02 ± 0.02 ^d,e^	0.03 ± 0.05 ^d,e^	1.66 ± 0.72 ^d,e^	109.75 ± 93.56 ^a,b,c,e^	23.84 ± 15.94 ^a,b,c,d^	<0.001
Bone (ng/g)							
	La	59.61 ± 14.23 ^d,e^	69.78 ± 10.67 ^d,e^	234.8 ± 102.49 ^d,e^	2017.35 ± 489.96 ^a,b,c,e^	1369.56 ± 177.08 ^a,b,c,d^	<0.001
	Ce	18.87 ± 8.25 ^d,e^	26.71 ± 9.27 ^d,e^	478.96 ± 268.6 ^d,e^	5418.33 ± 1369.7 ^a,b,c,e^	3463.71 ± 587.97 ^a,b,c,d^	<0.001
	Pr	21.86 ± 4.73 ^d,e^	23.95 ± 2.65 ^d,e^	81.93 ± 32.71 ^d,e^	825.33 ± 191.14 ^a,b,c,e^	558.15 ± 79.92 ^a,b,c,d^	<0.001
	Nd	3.54 ± 2.05 ^d,e^	3.82 ± 1.65 ^d,e^	29.33 ± 14.39 ^d,e^	348.55 ± 111.4 ^a,b,c,e^	235.26 ± 50.81 ^a,b,c,d^	<0.001
	Gd	0.86 ± 0.44 ^d,e^	0.75 ± 0.38 ^d,e^	6.86 ± 3.53 ^d,e^	87.43 ± 20.65 ^a,b,c,e^	58.16 ± 9.67 ^a,b,c,d^	<0.001
Hair (ng/g)							
	La	642.29 ± 277.15 ^d^	508.79 ± 211.18 ^d^	2160.79 ± 473.22 ^d^	18,320.41 ± 5757.4 ^a,b,c,e^	271.08 ± 210.62 ^d^	<0.001
	Ce	1377.46 ± 537.5 ^d^	1053 ± 396.25 ^d^	4086.98 ± 904.61 ^d^	32,629.22 ± 9544.6 ^a,b,c,e^	507.84 ± 404.31 ^d^	<0.001
	Pr	160.52 ± 61.74 ^d^	124.12 ± 47.2 ^d^	465.47 ± 104.41 ^d^	3523.33 ± 1035 ^a,b,c,e^	54.95 ± 43.41 ^d^	<0.001
	Nd	59.75 ± 27.99 ^d^	41.73 ± 15.06 ^d^	139.62 ± 27.19 ^d^	1096.23 ± 294.87 ^a,b,c,e^	27.29 ± 17.52 ^d^	<0.001
	Gd	18.1 ± 7.19 ^d^	13.74 ± 5.48 ^d^	60.87 ± 12.92 ^d^	462.43 ± 130.53 ^a,b,c,e^	6.78 ± 5.00 ^d^	<0.001
Liver(ng/g)							
	La	7.01 ± 2.53 ^d,e^	13.59 ± 6.35 ^d,e^	370.26 ± 204.61 ^d,e^	4552.18 ± 2141.21 ^a,b,c^	1272.52 ± 548.04 ^a,b,c^	<0.001
	Ce	12.59 ± 5.24 ^d,e^	23.39 ± 11.18 ^d,e^	785.92 ± 486.75 ^d,e^	12,807.13 ± 6105.85 ^a,b,c^	3723.99 ± 1610.61 ^a,b,c^	<0.001
	Pr	1.24 ± 0.67 ^d,e^	2.48 ± 1.28 ^d,e^	90.05 ± 55.61 ^d,e^	1617.66 ± 755.31 ^a,b,c^	453.3 ± 196.99 ^a,b,c^	<0.001
	Nd	1.54 ± 0.45 ^d,e^	1.35 ± 0.89 ^d,e^	31.45 ± 18.26 ^d,e^	617.95 ± 257.92 ^a,b,c^	174.86 ± 95.36 ^a,b,c^	<0.001
	Gd	0.28 ± 0.09 ^d,e^	0.34 ± 0.14 ^d,e^	9.86 ± 6.26 ^d,e^	165.12 ± 79.38 ^a,b,c,e^	39.46 ± 16.62 ^a,b,c,d^	<0.001

Note: Difference are significant when *p* < 0.05 level. ^a, b, c, d, e^ A significant difference with control, low-dose, medium-dose, high-dose, and detoxification, respectively.

**Table 2:** The relative element abundance of five REEs in different tissues and organs from rats in different groups.

		Control (n=10)	Low-Dose (n=10)	Medium-Dose (n=10)	High-Dose (n=10)	Detoxification (n=10)	*P-Value*
Whole Blood (%)							
	La	28.61 ± 1.27	27.38 ± 1.37	27.23 ± 1.15	29.06 ± 1.64	26.26 ± 4.16	0.052
	Ce	51.57 ± 1.9 ^d,e^	52.28 ± 3.02 ^d^	53.46 ± 1.35 ^d^	59.9 ± 1.64 ^a,b,c,e^	54.75 ± 3.48 ^a,d^	<0.001
	Pr	5.82 ± 0.41 ^d,e^	5.97 ± 0.31 ^d,e^	5.86 ± 0.52 ^d,e^	6.62 ± 0.33 ^a,b,c,e^	4.91 ± 0.77 ^a,b,c,d^	<0.001
	Nd	11.62 ± 2.46 ^d^	11.92 ± 3.43 ^d^	11.17 ± 1.61 ^d^	3.48 ± 0.96 ^a,b,c,e^	11.83 ± 2.4 ^d^	<0.001
	Gd	2.38 ± 0.49 ^d^	2.45 ± 0.78 ^d^	2.29 ± 0.48 ^d^	0.95 ± 0.12 ^a,b,c,e^	2.25 ± 0.61 ^d^	<0.001
Bone (%)							
	La	57.13 ± 4.46 ^c,d,e^	55.96 ± 7.34 ^c,d,e^	28.9 ± 1.75 ^a,b,d,e^	23.24 ± 0.52 ^a,b,c^	24.17 ± 0.81 ^a,b,c^	<0.001
	Ce	17.49 ± 5.15 ^b,c,d,e^	21.12 ± 6.15 ^a,c,d,e^	56.5 ± 2.72 ^a,b,d,e^	62.25 ± 0.67 ^a,b,c^	60.82 ± 1.43 ^a,b,c^	<0.001
	Pr	21.28 ± 3.52 ^b,c,d,e^	19.26 ± 2.05 ^a,c,d,e^	10.23 ± 1.11 ^a,b^	9.53 ± 0.28 ^a,b^	9.83 ± 0.18 ^a,b^	<0.001
	Nd	3.28 ± 1.49	3.06 ± 1.26	3.54 ± 0.14	3.98 ± 0.53	4.16 ± 0.79	0.081
	Gd	0.81 ± 0.34 ^b^	0.6 ± 0.29 ^a,c,d,e^	0.82 ± 0.04 ^b^	1.01 ± 0.03 ^b^	1.02 ± 0.04 ^b^	<0.001
Hair (%)							
	La	28.06 ± 1.54 ^c,d,e^	28.76 ± 1.59 ^c,d,e^	31.25 ± 0.61 ^a,b,d^	32.62 ± 1.06 ^a,b,c,e^	31.25 ± 0.56 ^a,b,d^	<0.001
	Ce	61.38 ± 1.33 ^c,d,e^	60.85 ± 1.41 ^c,d,e^	59.08 ± 0.59 ^a,b^	58.29 ± 0.87 ^a,b^	57.9 ± 1.22 ^a,b^	<0.001
	Pr	7.17 ± 0.3 ^c,d,e^	7.16 ± 0.11 ^c,d,e^	6.72 ± 0.12 ^a,b,d,e^	6.29 ± 0.18 ^a,b,c^	6.3 ± 0.21 ^a,b,c^	<0.001
	Nd	2.58 ± 0.23 ^e^	2.45 ± 0.25 ^e^	2.06 ± 0.37 ^e^	1.98 ± 0.13 ^e^	3.71 ± 1.52 ^a,b,c,d^	<0.001
	Gd	0.8 ± 0.03	0.79 ± 0.03	0.88 ± 0.04	0.83 ± 0.04	0.84 ± 0.19	0.191
Spleen (%)							
	La	45.91 ± 10.09 ^c,d,e^	41.42 ± 8.57 ^c,d,e^	26.4 ± 1.29 ^a,b,d^	81.29 ± 1.19 ^a,b,c,e^	21.52 ± 2.74 ^a,b,d^	<0.001
	Ce	36.37 ± 18.23 ^c,d,e^	40.06 ± 10.74 ^c,d,e^	61.49 ± 1.04 ^a,b,d^	10.62 ± 0.18 ^a,b,c,e^	65.8 ± 2.15 ^a,b,d^	<0.001
	Pr	9.47 ± 1.75 ^c,d^	8.71 ± 0.99 ^d^	8.06 ± 0.24 ^a,d^	4.19 ± 0.43 ^a,b,c,e^	8.66 ± 0.48 ^d^	<0.001
	Nd	7.57 ± 8.02	9.53 ± 3.42 ^c,d,e^	3.39 ± 0.59 ^b^	3.07 ± 1.08 ^b^	3.32 ± 0.4 ^b^	<0.001
	Gd	0.67 ± 0.99	0.29 ± 0.44	0.66 ± 0.07	0.83 ± 0.06	0.7 ± 0.05	0.158
Liver (%)							
	La	31.22 ± 1.3 ^b,c,d,e^	33.13 ± 1.95 ^a,c,d,e^	29.1 ± 0.92 ^a,b,d,e^	23.09 ± 1.09 ^a,b,c^	22.46 ± 1.37 ^a,b,c^	<0.001
	Ce	55.16 ± 1.84 ^c,d,e^	56.44 ± 2.24 ^c,d,e^	60.74 ± 0.87 ^a,b,d,e^	64.66 ± 0.97 ^a,b,c^	65.51 ± 1.39 ^a,b,c^	<0.001
	Pr	5.24 ± 0.68 ^b,c,d,e^	5.87 ± 0.5 ^a,c,d,e^	6.96 ± 0.12 ^a,b,d,e^	8.2 ± 0.18 ^a,b,c^	7.97 ± 0.24 ^a,b,c^	<0.001
	Nd	7.11 ± 1.67 ^b,c,d,e^	3.71 ± 3.43 ^a^	2.44 ± 0.28 ^a^	3.21 ± 0.32 ^a^	3.35 ± 1.22 ^a^	<0.001
	Gd	1.26 ± 0.19 ^b,c,d,e^	0.85 ± 0.19 ^a,e^	0.76 ± 0.04 ^a^	0.83 ± 0.03 ^a^	0.7 ± 0.04 ^a,b^	<0.001

Note: Difference are significant when *p* < 0.05 level. ^a, b, c, d, e^ A significant difference with control, low-dose, medium-dose, high-dose, and detoxification, respectively.

**Table 3:** The correlations of REE concentrations among different tissues and organs of rats.

Variables	La				Ce				Nd				Gd				Pr			
	Whole Blood	Bone	Hair	Spleen	Whole Blood	Bone	Hair	Spleen	Whole Blood	Bone	Hair	Spleen	Whole Blood	Bone	Hair	Spleen	Whole Blood	Bone	Hair	Spleen
**Bone**	0.095				0.583 *				−0.022				−0.212				0.053			
**Hair**	0.039	−0.791 *			−0.533 *	-0.760 *			0.445 *	0.178			0.090	0.158			0.066	0.790 *		
**Spleen**	0.380 *	−0.044	0.180		−0.414 *	0.124	−0.154		0.155	0.150	−0.004		−0.202	0.708 *	0.089		−0.451 *	0.461 *	0.575 *	
**Liver**	0.092	0.849 *	−0.680 ^*^	−0.184	0.578 *	0.877 *	−0.745 ^*^	0.060	0.085	−0.240	0.089	0.131	0.185	−0.252	−0.093	−0.104	−0.021	−0.817 ^*^	−0.822 *	−0.567 *

Note: * *p* < 0.05.

## Supplementary Materials

The following are available online at https://www.mdpi.com/1660-4601/17/4/1399/s1, Table S1: The contents of REEs in the food and the rare earth citrate.
