# Supplementary material for: The Accumulation and Metabolism Characteristics of Rare Earth Elements in Sprague–Dawley Rats
Source: Int J Environ Res Public Health. 2020 Feb 21;17(4):1399. doi: 10.3390/ijerph17041399 (PMC7068551; doi:10.3390/ijerph17041399)
Supplement: Supplementary file 1 [file ijerph-17-01399-s001.pdf]

International Journal of
Environmental Research and Public Health

Table S1. The contents of REEs in the food and the rare earth citrate.

Variables	Food (ng/g)	Rare earth citrate (ng/g）	Rare earth citrate / Food Ratio
La	157.00	69669963.37	443757.7
Ce	289.00	154846520.10	535801.10
Pr	39.00	17421611.72	446708.00
Nd	143.00	11027655.68	77116.47
Gd	33.00	2210989.01	66999.67
